# Fellow travellers: a concordance of colonization patterns between mice and men in the North Atlantic region

**DOI:** 10.1186/1471-2148-12-35

**Published:** 2012-03-19

**Authors:** EP Jones, K Skirnisson, TH McGovern, MTP Gilbert, E Willerslev, JB Searle

**Affiliations:** 1 Department of Biology, University of York, PO Box 373, York YO10 5YW, UK; 2 Population Biology and Conservation Biology, Evolutionary Biology Centre, University of Uppsala, Norbyvägen 18 D, SE-752 36 Uppsala, Sweden; 3 Laboratory of Parasitology, Institute for Experimental Pathology, University of Iceland, Keldur, 112, Reykjavík, Iceland; 4 Anthropology Department, Hunter College CUNY, 695 Park Avenue, New York City 10021, NY, USA; 5 Centre for GeoGenetics, Natural History Museum of Denmark, University of Copenhagen, Øster Voldgade 5-7, 1350 Copenhagen, Denmark; 6 Department of Ecology and Evolutionary Biology, Cornell University, Corson Hall, Ithaca, NY 14853-2701, USA

## Abstract

**Background:**

House mice (*Mus musculus*) are commensals of humans and therefore their phylogeography can reflect human colonization and settlement patterns. Previous studies have linked the distribution of house mouse mitochondrial (mt) DNA clades to areas formerly occupied by the Norwegian Vikings in Norway and the British Isles. Norwegian Viking activity also extended further westwards in the North Atlantic with the settlement of Iceland, short-lived colonies in Greenland and a fleeting colony in Newfoundland in 1000 AD. Here we investigate whether house mouse mtDNA sequences reflect human history in these other regions as well.

**Results:**

House mice samples from Iceland, whether from archaeological Viking Age material or from modern-day specimens, had an identical mtDNA haplotype to the clade previously linked with Norwegian Vikings. From mtDNA and microsatellite data, the modern-day Icelandic mice also share the low genetic diversity shown by their human hosts on Iceland. Viking Age mice from Greenland had an mtDNA haplotype deriving from the Icelandic haplotype, but the modern-day Greenlandic mice belong to an entirely different mtDNA clade. We found no genetic association between modern Newfoundland mice and the Icelandic/ancient Greenlandic mice (no ancient Newfoundland mice were available). The modern day Icelandic and Newfoundland mice belong to the subspecies *M. m. domesticus*, the Greenlandic mice to *M. m. musculus*.

**Conclusions:**

In the North Atlantic region, human settlement history over a thousand years is reflected remarkably by the mtDNA phylogeny of house mice. In Iceland, the mtDNA data show the arrival and continuity of the house mouse population to the present day, while in Greenland the data suggest the arrival, subsequent extinction and recolonization of house mice - in both places mirroring the history of the European human host populations. If house mice arrived in Newfoundland with the Viking settlers at all, then, like the humans, their presence was also fleeting and left no genetic trace. The continuity of mtDNA haplotype in Iceland over 1000 years illustrates that mtDNA can retain the signature of the ancestral house mouse founders. We also show that, in terms of genetic variability, house mouse populations may also track their host human populations.

## Background

During the Viking Age (late 8th to mid-10th C), sections of the northern and western British Isles (northern Scotland, the Scottish Isles, Isle of Man and large portions of Ireland) had extensive contact with Norway, including colonization by Norwegian settlers [1,2]. These Norwegian Vikings also explored across the North Atlantic, discovering and creating settlements in the Faroe Islands, Iceland, Newfoundland and Greenland. They intentionally brought with them a variety of domesticated animals but they also would have unintentionally brought pest or commensal species, including the house mouse (*Mus musculus*). Several previous phylogeographic studies have traced the link between house mice and Norwegian Viking-associated regions in continental Europe or offshore, with the key finding that a particular house mouse mitochondrial DNA (mtDNA) clade is found in these areas of Scotland, Ireland and Norway [3-5]. Here we extend these studies on house mice to other areas settled and explored by the Norwegian Vikings (Iceland, Greenland, Newfoundland). An assumption made in previous similar phylogeographic studies is that the current populations of house mice reflect the historical patterns [6] and that the founding population and routes of colonization can be inferred from the modern population. One way to test this assumption is to use ancient DNA to sample the original population, an approach we use here.

Given that the house mouse niche was created by humans, it has been suggested that demographic changes in humans can lead to similar changes in house mice and that these can leave a discernable trace in the population genetics of both species [7]. In the case of the house mouse population genetics, a recent dip in effective population size of the western house mouse subspecies reconstructed from Bayesian skyline plots might be linked to the modernization of agriculture [8], while, at the local scale, house mouse genetic diversity on islands within an archipelago is positively correlated to human population size [9]. We further investigate this association between human and mouse population genetics here.

**Figure 1:**
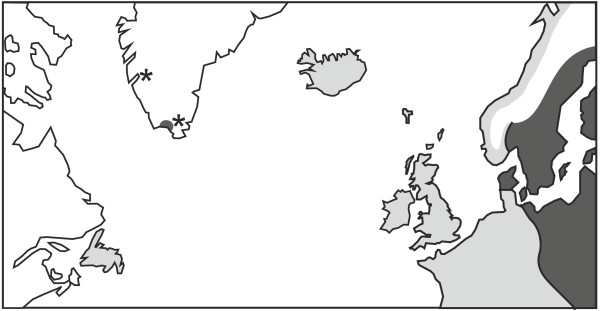
**Distribution of *M. m. musculus *(dark shaded) and *M. m. domesticus *(lighter shaded) in northern Europe and the North Atlantic region from previously published data and data collected here (Iceland, Greenland, Newfoundland)**. Areas with no shading either have no house mice or the subspecies distribution is unknown, and areas with some admixture of the two are shaded according to the dominant subspecies. The locations marked with asterisks are sites in Greenland represented by ancient DNA samples. From the mtDNA haplotypes, these were *M. m. domesticus *derived from the Icelandic population.

Two subspecies of house mice are present in the potential source areas for the house mice in Iceland, Greenland and Newfoundland: *M. m. domesticus *from the western section of northern Europe (e.g. France, Belgium, the Netherlands, the British Isles, parts of western Germany) and *M. m. musculus *from the eastern section of northern Europe (e.g. eastern Germany, Denmark and Sweden and eastwards from there) (Figure 1; for a full distribution, see [5,10]). Therefore, we used four nuclear molecular markers which reliably discriminate between the two subspecies using fixed polymorphisms (two autosomal and one from each sex chromosome [3]). MtDNA sequence data are not a fully reliable indication of the subspecies, as introgression of *M. m. domesticus*-type mtDNA into otherwise *M. m. musculus *mice is widespread in Sweden and Finland [5,11].

Our findings were as follows: from mtDNA data, house mice arrived in Iceland from either Norway or the northern section of the British Isles in the 10^th ^C, and from there were transported to the two Viking settlements in Greenland. The mice in Iceland have persisted to the present day, while those in Greenland apparently became extinct and subsequently recolonized more recently, likely from Denmark. We found no evidence that the mice were brought to Newfoundland in the Viking period. The Icelandic house mouse populations share the low genetic diversity of the human population. All the mice we studied were *M. m. domesticus*, except the late-colonizing individuals on Greenland, which were *M. m. musculus*, in support of their Danish origin.

## Methods

### Samples

**Figure 2:**
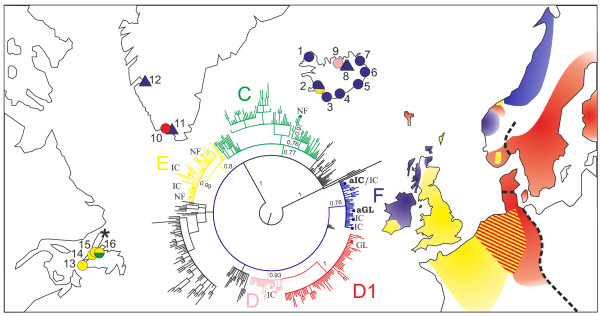
**Collection localities for the house mice used in this study and the phylogenetic tree based on their mtDNA sequences and additional published material (see Methods section)**. Our new collection localities are shown as triangles (ancient specimens) or circles (modern specimens) and are coloured according to the mtDNA clade affinity, with the clade name printed in the same colour next to the clade; previously published haplotype distribution (approximate) is shaded. Location numbers on the map correspond to those used in Table 1. New haplotypes are indicated on the tree as squares and labelled according to their location (NF = Newfoundland, GL = Greenland, IC = Iceland). Posterior probabilities of 0.7 or above are shown for the branches leading to haplotypes found in this study. Ancient, mostly Viking Age, haplotypes are shown in bold (aIC = ancient Iceland, aGL = ancient Greenland). The Viking Age site in Newfoundland is marked with *. The *M. m. musculus/M. m. domesticus *hybrid zone is marked with a thick dashed black line.

Modern house mouse populations were sampled across Iceland (9 localities), at Narsaq in Greenland (near the Viking Age 'Eastern Settlement') and in the north-west of Newfoundland (near the Norwegian Viking archaeological site at L'Anse aux Meadows [12], 4 localities) (Figure 2 and Table 1). Ancient DNA was obtained from archaeological house mouse bones. In Greenland, these were from the Norwegian Viking 'Eastern Settlement' (3 individuals) and 'Western Settlement' (2 individuals) [13,14], dating from between 1015-1165 AD. In Iceland, these were from four archaeological sites in the north of Iceland, three of which date to the 10th C (1 individual per site) [15-17] and one of which dates to the Medieval period or later (1477-1717 AD; 2 individuals; Table 1) [18].

**Table 1:** Details of the modern and ancient (mostly Viking Age) samples of house mouse used in this study.

Map ref	Location	Country	N (subsp)	N (mt)	Year (AD)	mt clade	mtDNA haplotype
1	Bolungarvik	Iceland	3	3	2003-2006	F	U47436

2	Mosfellsbær	Iceland	3	3	2003	F	U47436

2	Keldur Institute, Reykjavik	Iceland	2	5	1992	F	U47436

2	Kópavogur	Iceland	3	3	1998-2005	F	U47436

2	Öldugrof, Reykjavik	Iceland	1	2	2004	E	U47430

2	Smidshofdi, Reykjavik	Iceland	1	1	2004	F	U47436

2	Breidholt, Reykjavik	Iceland	1	1	2004	F	IRB1

2	Grafarvogur, Reykjavik	Iceland	1	2	2003-2004	F	U47436

2	Arbær, Reykjavik	Iceland	1	1	2004	E	IRA1

2	Naustabryggja, Reykjavik	Iceland	1	1	2004	F	U47436

3	Storhofdi Lighthouse, Westman Islands	Iceland	3	9	1996	F	U47436, IRB1

3	Heimaey, Westman Islands	Iceland	3	4	1992-2006	F	U47436

4	Kalfafell, Sudursveit	Iceland	3	4	2005	F	U47436

5	Smyrlabjorg, Sudursveit	Iceland	-	3	? Modern	F	U47436

6	Reydarfjordur	Iceland	3	5	2005-2006	F	U47436

7	Asbrandsstadir, Vopnafjordur	Iceland	3	5	2005	F	IAs1

9	Sveinbjarnargerdi, Svalbardsstrond	Iceland	4	4	2006	D	ISv1

10	Narsaq	Greenland	2	2	2010	D1	U47455

13	Saint Andrew's, Codroy, Newfoundland	Canada	6	4	2008	E	U47431

13	Searston, Codroy, Newfoundland	Canada	5	5	2008	E	U47431

14	Hammond Farm, Steady Brook, Newfoundland	Canada	6	6	2008	E	NLPB1

15	Pynn's Brook, Newfoundland	Canada	1	1	2008	E	NLPB1

16	Site 1, Cormack, Newfoundland	Canada	5	6	2008	E,C	U47431, NLCo1

16	Site 2, Cormack, Newfoundland	Canada	6	5	2008	E,C	U47431, NLCo1

8	Hofstaðir, Myvatnssveit^1^	Iceland	-	1	940	F	U47436

8	Skutustadir, Myvatnssveit^2^	Iceland	-	2	1477-1717	F	U47436

8	Hrísheimar, Myvatnssveit^3^	Iceland	-	1	871-940	F	U47436

8	Sveigakot, Myvatnssveit^4^	Iceland	-	1	900-1000	F	U47436

11	Farm Beneath the Sand (old Western Settlement)^5^	Greenland	-	2	1110-1150	F	AnctGL

12	Vatnahverfi (old Eastern Settlement)^6^	Greenland	-	3	1015-1165	F	AnctGL

'N (subsp)' is the number of samples tested for the subspecies specific markers, 'N (mt)' is the number of mtDNA sequences obtained. Haplotype names in the format 'U474xx' are from [11], all others are new for this study. All are archived in GenBank.

^1 ^Dated from tephra layers [16].

^2 ^Dated from context [18].

^3 ^Dated from tephra layers [15].

^4 ^Dated from context [17].

^5 ^Dated from AMS ^14 ^C data [14].

^6 ^Dated from context [13].

### DNA extraction, amplification and sequencing

Modern DNA samples were extracted using Qiagen Blood and Tissue kits. The mtDNA control region and adjacent tRNAs were amplified by PCR using the primer pair H2228 (TTA TAA GGC CAG GAC CAA AC) and L15774 (TGA ATT GGA GGA CAA CCA GT) [4], and resulting sequences shortened to positions 15424-16276 of Bibb et al. [19] to align with previously published house mouse sequences [all sequences included in 3,5,9]. Nucleotide diversity (π) and haplotype diversity (Hd) were calculated using DNAsp v. 4.20.2 [20].

For the ancient DNA samples, whole pulverised femurs were incubated in 1.5 ml 0.5 M EDTA pH 8 on a shaking platform for 12 h and the resulting solution spun through a Centricon tube until 200 μl of liquid remained. This concentrated liquid was then purified and cleaned using the Qiagen QIAQuick kit [21]. All extraction steps and PCR set up was done in a dedicated ancient DNA facility, away from any PCR or post-PCR areas. The D-loop and flanking sequences were amplified in 8 or 9 overlapping 112 to 222 bp fragments (details in Additional file 1: Table S1) and sequenced using the PCR primers. The most variable section (between positions 15381 and 15663 [19]) was cloned a minimum of 8 times for each sequence to check for potential contamination or incorrect base calls.

The alignment of the sequences published here and previously published sequences [3,5,9] were used to create a Bayesian inference phylogenetic tree with MrBayes [22], using the nucleotide substitution model and gamma distribution rates selected by jModelTest [23]. MrBayes was run for 10 million iterations with two sets of five chains with a 0.05 incremental heating parameter, with a burn-in and convergence estimated using PSRF statistics. The tree was visualized in FigTree v.1.3.1. and clades assigned and named following a previous nomenclature [3].

Representative mice (Table 1) were scored for four markers with fixed mutational differences between the subspecies *M. m. musculus *and *M. m. domesticus*: Abpa on chromosome 7, D11 cenB2 on chromosome 11, Zfy2 on the Y chromosome and Btk on the X chromosome. Screening involved either allele specific PCR (Abpa, D11 cenB2) or PCR amplification and scoring the fixed allele size differences revealed by gel electrophoresis (Zfy2, Btk). Full references and methods are given in [3].

### Microsatellite amplification, scoring and analysis

Where larger population samples were available (n = 7 to 16; Table 2) the modern Icelandic mice were typed at 14 nuclear microsatellite loci: D1Mit64, D2Mit1, D3Mit117, D4Mit103, D5Mit145, D8Mit58, D9Mit218, D10Mit188, D12Mit145, D13Mit153, D15Mit12, D16Mit2, D17Mit19 and D19Mit150 (primer details at http://www.ncbi.nlm.nih.gov/). Each microsatellite was amplified by PCR using a simple forward primer and a fluorescent-labelled reverse primer with Qiagen Multiplex PCR Kits. Fragment sizes were scored on an ABI 3130 Genetic Analyzer (Applied Biosystems) and alleles assigned using GeneMapper v.3.7.

**Table 2:** Summary population genetic statistics for the Icelandic house mouse populations, based on the microsatellite data.

Location	N	A	SD	Ho	SD	He	SD
Bolungarvik	11	2.08	0.79	0.24	0.21	0.29	0.25

Grafarvogur, Reykjavik	12	2.33	0.65	0.43	0.26	0.42	0.18

Storhofdi Lighthouse	15	2.83	1.03	0.32	0.23	0.39	0.22

Kalfafell	7	1.25	0.45	0.14	0.30	0.12	0.22

Reydarfjordur	16	2.17	0.94	0.25	0.26	0.26	0.23

Asbrandsstadir	12	2.67	0.78	0.24	0.18	0.34	0.16

Sveinbjarnargerdi	8	3.50	1.17	0.36	0.18	0.47	0.15

*Total/mean*	*81*	*2.40*	*0.83*	*0.28*	*0.23*	*0.33*	*0.20*

N = number of samples, A = mean number of alleles per locus, SD = standard deviation, Ho = observed heterozygosity, He = expected heterozygosity.

The mean number of microsatellite alleles per locus, observed heterozygosity [24] and expected heterozygosity [25] were calculated per population using the POPGENE program v1.32 [26]. Deviations from Hardy-Weinburg equilibrium per locus and per population were assessed using GENEPOP [27]; run parameters were 5000 batches of 20000 iterations.

## Results and discussion

### Subspecies identity of modern populations

Using the four nuclear markers to distinguish the subspecies, the Icelandic and Newfoundland populations were entirely *M. m. domesticus*-like, with the exception of one mouse from Reykjavik which was *musculus*-like for the *Zfy2 *marker on the Y-chromosome. In contrast, the modern Greenlandic mice were *musculus*-like for the four nuclear markers.

### mtDNA phylogeography

Within Iceland, the majority of the modern house mouse sequences and all the ancient sequences belonged to a lineage (referred to as clade F [3]) that previous studies showed to be restricted almost exclusively to areas with a Norwegian Viking history (i.e. Northern Scotland and the Scottish isles, Norway, Ireland and the Isle of Man [3-5]). Given that Iceland was also colonized by Norwegian Vikings, this previously-shown close fit of mouse and human history has been maintained. The haplotype of the four ancient DNA samples was the same as the most common modern haplotype on Iceland, U47436 (haplotype first identified in [11]; Figure 2), showing a continuity of the Icelandic house mouse population from the initial colonization period (between 874 and 930 AD), though medieval times and into the present. This is in agreement with previous suggestions that house mouse populations are resistant to subsequent invasion by later arrivals [3,4,6], and provides support for other studies which have assumed that modern house mouse phylogeography reflects ancient events.

There was insufficient resolution in the sequence data to identify the source of the Icelandic house mouse population with any precision, as the most common haplotype (U47436) has been found widely in northwestern Europe. This haplotype occurs in the north of mainland Scotland and Orkney, and is widespread in Ireland and western Norway (including Trondheim, from where the majority of early seafaring to Iceland started out [28]). The mice might have been introduced (presumably as stowaways on Viking cargo boats) direct from Norway, but might also come from the far north or west of the British Isles. Genetic studies on modern day human populations have found that approximately 80% of the Icelandic patrilineal ancestry is of Scandinavian origin [29], while only around 37% of the matrilineal ancestry is Scandinavian, the majority of the remainder having a British or Irish origin [2], fitting both the possible sources of the house mouse populations. Future studies may be able to determine more accurately the origin of the early house mouse colonists and, by proxy, provide a remarkable new perspective on early human colonization of Iceland. This may be achieved by using mouse sequence data with better resolution, e.g. whole mtDNA genomes.

The five ancient Greenlandic sequences have a single haplotype that is one nucleotide different from the widespread Icelandic haplotype U47436, consistent with the Viking Age Greenlandic mice deriving from the Icelandic population (Figure 2). However, the modern mice sampled in Narsaq, Greenland (n = 2) belong to a distinctly different mtDNA clade to that found in Iceland and are from a different subspecies, *M. m. musculus*, showing a lack of genetic continuity between the sampled ancient and modern Greenlandic mouse populations. Like the Norwegian Viking human population of Greenland, which disappeared around 1450 AD, the Viking Age mice of Greenland seemingly failed to persist to the modern day. Although the mice currently found in Narsaq are otherwise *M. m. musculus*, they carry mtDNA of a *M. m. domesticus *type. This situation is very similar to that in northern Denmark and in Sweden, where the house mice are *M. m. musculus*-like morphologically and for nuclear markers, but carry a *M. m. domesticus *type mtDNA [11]. The mtDNA found in Sweden and northern Denmark belongs to the same clade (D1) as that found in modern Greenlandic mice and indeed the specific Greenlandic haplotype is widespread in both countries [5,11]. Therefore, it appears likely that the house mice in Greenland were recently reintroduced from Denmark, accounting for their *musculus *morphology and nuclear genome and *domesticus *clade D1 mtDNA. This is also consistent with modern Greenlandic history which came under Danish governance in the 1500s, becoming host to greater contact with Denmark from the 1700s.

There were no archaeological mouse samples from which we could derive ancient DNA at L'Anse aux Meadows in Newfoundland and we can only speculate whether house mice arrived in the Viking period at all. Although the settlement is only thought to have persisted for a very short time (at around 1000 AD), leaving a brief window for the mice to arrive, we know that house mice did arrive early with Norwegian Vikings in Iceland, based on the archaeological mouse specimens there, and may have arrived similarly early in Greenland and Newfoundland. Had the mice been introduced onto Newfoundland, it is uncertain whether they would have been able to survive in a free-living state on the island when the Viking people left; in some situations house mice are able to form long-term outdoor populations and in others they are not [6,30]. Other (non-European) human cultures were present in Newfoundland at the time [31], but their more nomadic settlement pattern seems unlikely to have provided house mice with suitable habitat. The modern mouse samples from Newfoundland derive from two clades not found in Iceland, showing that they are not descended from a remnant population brought in by Norwegian Vikings. The majority of the mice belong to clade E, mostly from a single haplotype (U47431, n = 21) which is widespread in Britain [3,4] (Figure 2). Clade E is globally common, found in Northern Europe, Mauritania, Cameroon, Morocco and New Zealand. As has been discussed elsewhere [32], this clade of mice was perfectly placed to take advantage of the large movements of people and goods that occurred during the expansion of the European empires in the 18th century, and the presence of the clade in Newfoundland is likely to be an early product of this, either direct from Britain or via mainland Canada.

The other clade we found in Newfoundland is clade C, also found around the Mediterranean Basin, the Spanish and Portuguese Atlantic coasts, locations in Africa and two sites in the United States. The Newfoundland haplotype is very similar to two of the haplotypes previously found in the United States, with which they may share a common derivation from Mediterranean Europe.

### Genetic diversity

Like the Icelandic human population [33], the Icelandic house mice have low mtDNA genetic diversity (Hd = 0.479, π = 0.00292 -/+ 0.0009). We also assessed the house mouse genetic diversity in Iceland using 14 microsatellite markers. The mean number of alleles, observed and unbiased expected heterozygosity values for the Icelandic mouse populations (Table 2) are much lower than typically found for microsatellites in other wild mouse populations [34,35]. This shared reduced genetic diversity between Icelandic mice and men is likely to have similar causes: founder events and genetic drift. The Newfoundland house mouse populations also had low mtDNA genetic diversity (Hd = 0.542, π = 0.00223-/+ 0.0009), likely because of their relative isolation and even more recent origin than the Icelandic mice.

### The history of the house mouse in the North Atlantic

**Figure 3:**
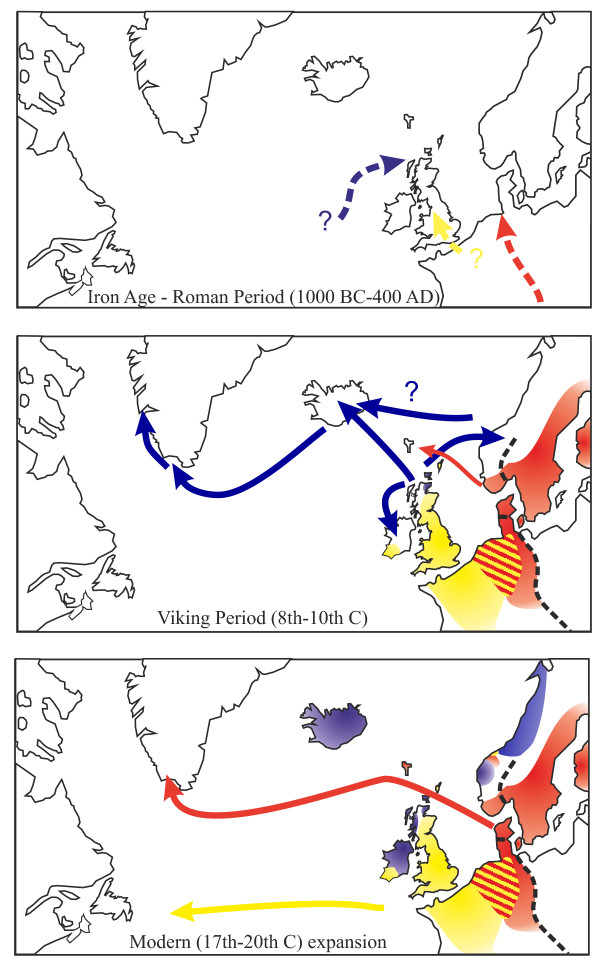
**Expansion of the house mouse in the North Atlantic area in the Iron Age - Roman period, Viking Period and 'Modern' era based on mtDNA sequences**. Colours for the mtDNA clades are the same as in Figure 2, which depicts the current distribution of the clades. The *M. m. musculus/M. m. domesticus *hybrid zone is marked with a black line. The mice that originally colonized Greenland in the Viking era may have gone extinct; only mice of the Danish colonization have been found there now.

Using the data from the distribution of the house mouse mtDNA clades from both ancient and modern DNA, it is possible to make an overall synthesis of the colonization history of the house mouse (both *M. m. domesticus *and *M. m. musculus*) in the North Atlantic region, extending far beyond earlier attempts based on morphology and allozymes [36,37]. In conjunction with other sources of evidence (historical, archaeological), we relate this to the patterns of human colonization and movement (Figure 3).

Expanding their range from the Mediterranean region, the initial northwest-wards spread of *M. m. domesticus *most likely occurred in the Iron Age and into the Roman period [38,39], concurrent with the expansion of the house mouse niche (larger settlements) and increasing human movements. Evidence for this comes from the zooarchaeological record [3], and phylogeographic data can be interpreted in this context [3,7]. At the end of this period, *M. m. domesticus *would have become established across much of north-western continental Europe and Britain.

The next extensive movements of *M. m. domesticus *in the North Atlantic region came in the Viking period, during which mice from clade F were dispersed by the Norwegian Vikings perhaps initially from the Scottish isles [4], to Norway [4,5], Ireland [3,4], Iceland and Greenland (this study). Given the earlier settlements in the northern Scottish Isles and their more extensive cultural contacts (discussed in [3,4]), it appears likely the Norwegian Vikings inadvertently brought house mice from the Scottish Isles to Norway, rather than vice versa. This expansion likely reflects increased urbanization (and therefore increased mouse habitat) in Norway and Ireland at this time [1], as well as the first human settlement of Iceland by Norwegian Vikings. One striking finding is that the Faroe Islands, also first colonized by the Norwegian Vikings, have mice of clade D1 rather than clade F [9]. Clade D1 is well-represented in southern Norway ([5], Figures 2 and 3) but our results indicate that the process of mouse, and therefore human, colonization of Faroe (likely from southern Norway, consistent with human Y-chromosome ancestry [40]) and Iceland (from western Norway or the British Isles) were distinctly different, despite sometimes being grouped under the homogenous umbrella of "Norwegian Viking colonization".

The final colonization of new space by house mice in the North Atlantic region occurred during the modern period with the rediscovery and colonization of North America by European people from the 15th century onwards. *M. m. domesticus *of clade E were apparently introduced from Britain to Newfoundland, and likely to the North American continent beyond. *M. m. musculus *were also able to establish overseas, with populations of these mice seemingly carried from Danish ports to Greenland and reflected in the modern day populations.

## Conclusions

The inadvertent spread of house mice by Europeans in the North Atlantic region has left a living artefact of human colonization, expansion, local extinction and recolonization which can be traced using both phylogeography and population genetics. In this region, house mice are a culturally specific marker restricted to movement of European people. They are sufficiently dependent on their human host populations that house mouse demography as well as movements may mirror that of the human population. Using ancient DNA, we have conclusively demonstrated that those ancient colonization events are reflected by the modern phylogeography in house mice.

## Authors' contributions

EPJ and JBS conceived of the study. EPJ carried out the molecular work and analysis and wrote the paper together with JBS. EPJ, KS and THMcG designed sampling strategies and obtained the Newfoundland, Icelandic and ancient samples, respectively. MTBG and EW substantially assisted with the ancient DNA design and methods. All authors read and approved the final manuscript.

## Supplementary Material

Additional file 1 **Table S1**. Primers used to amplify the ancient mtDNA D-loop sequence. Primers in italics were used to amplify a smaller fragment where the first set of primers for that region failed to amplify; in one case (Frag 2) the fragment was sub-divided into two reactions. Fragments marked with an asterisk were cloned. Click here for file
