# Supplementary material for: Fellow travellers: a concordance of colonization patterns between mice and men in the North Atlantic region
Source: BMC Evol Biol. 2012 Mar 19;12:35. doi: 10.1186/1471-2148-12-35 (PMC3315747; doi:10.1186/1471-2148-12-35)
Supplement: Additional file 1 — Table S1. Primers used to amplify the ancient mtDNA D-loop sequence. Primers in italics were used to amplify a smaller fragment where the first set of primers for that region failed to amplify; in one case (Frag 2) the fragment was sub-divided into two reactions. Fragments marked with an asterisk were cloned. [file 1471-2148-12-35-S1.DOC]

**Supplementary material**

Table S1. Primers used to amplify the ancient mtDNA D-loop sequence. Primers in italics were used to amplify a smaller fragment where the first set of primers for that region failed to amplify; in one case (Frag 2) the fragment was sub-divided into two reactions. Fragments marked with an asterisk were cloned.

| Fragment name | Primer name | Sequence 5' to 3' | Fragment length |
| --- | --- | --- | --- |
| Frag 1 * | L15380 | GCACCCAAAGCTGGTATTCT | 149 |
|  | H15528 | TTTTATGACCTGAACCATTGAYT |  |
| Frag 2 * | L15450 | TATGTATATCGTACATTAAAYTAT | 219 |
|  | H15671 | GAAGGGGATAGTCATATGGAAG |  |
| *Frag 2a ** | *L15480* | *CCAAGCATATAAGCAAGTACAT* | *179* |
|  | *H15659* | *CATATGGAAGAGAAGAGTTTATG* |  |
| *Frag 2b ** | *L15602* | *ATATCTGTGTTATCTGACATAC* | *138* |
|  | *H15740* | *AGAAGAGGGGCATWGGTGG* |  |
| Frag 3 | L15614 | TTATCTGACATACACCATACAG | 222 |
|  | H15835 | TATGGGCGATAACGCATTTGAT |  |
| *or* | *L15689* | *TCTACCATCCTCCGTGAAAC* | *147* |
| Frag 4 | L15780 | CTTTATCAGACATCTGGTTCTT | 209 |
|  | H15989 | GCGTCTAGACTGTGTGCTGT |  |
| *or* | *L15842* | *CCTTAAATAAGACATCTCGATG* | *147* |
| Frag 5 | L15937 | CTTTCATCAACATAGCCGTCAA | 215 |
|  | H16195 | TGTTTTTGGGGTTTGGCATTAA |  |
| *Frag 5a* | *L15990* | *CACCTACGGTGAAGAATCATT* | *150* |
|  | *H16139* | *GGTTTGGCATTAAGAGGAGG* |  |
| Frag 6 | L16041 | TATTCATGCTTGTTAGACATAAA | 112 |
|  | H16195 | TGTTTTTGGGGTTTGGCATTAA |  |
| Frag 7 | L16069 | CTCAATACCAAATTTTAACTCTC | 160 |
|  | H16229 | GTCATATTTTGGGAACTACTAG |  |
| Frag 8 | L16181 | CTATCAAACCCTATGTCCTGA | 144 |
|  | H16325 | CTTGTTAATGTTTATTGCGTAAT |  |
